# Flight performance of actively foraging honey bees is reduced by a common pathogen

**DOI:** 10.1111/1758-2229.12434

**Published:** 2016-07-07

**Authors:** Trish Wells, Stephan Wolf, Elizabeth Nicholls, Helga Groll, Ka S. Lim, Suzanne J. Clark, Jennifer Swain, Juliet L. Osborne, Alison J. Haughton

**Affiliations:** ^1^ Rothamsted ResearchHarpendenUK; ^2^ Present address: School of Biological and Chemical SciencesQueen Mary University of LondonLondonUK; ^3^ Present address: School of Life SciencesUniversity of SussexBrightonUK; ^4^ Present address: PPD, Granta Park, Great AbingtonCambridgeUK; ^5^ Present address: Environment and Sustainability InstituteUniversity of ExeterPenrynUK

## Abstract

Sudden and severe declines in honey bee (*Apis mellifera*) colony health in the US and Europe have been attributed, in part, to emergent microbial pathogens, however, the mechanisms behind the impact are unclear. Using roundabout flight mills, we measured the flight distance and duration of actively foraging, healthy‐looking honey bees sampled from standard colonies, before quantifying the level of infection by *Nosema ceranae* and Deformed Wing Virus complex (DWV) for each bee. Neither the presence nor the quantity of *N*. *ceranae* were at low, natural levels of infection had any effect on flight distance or duration, but presence of DWV reduced flight distance by two thirds and duration by one half. Quantity of DWV was shown to have a significant, but weakly positive relation with flight distance and duration, however, the low amount of variation that was accounted for suggests further investigation by dose‐response assays is required. We conclude that widespread, naturally occurring levels of infection by DWV weaken the flight ability of honey bees and high levels of within‐colony prevalence are likely to reduce efficiency and increase the cost of resource acquisition. Predictions of implications of pathogens on colony health and function should take account of sublethal effects on flight performance.

## Introduction

Decadal and on‐going declines in the number of colonies of managed honey bees in the USA and Europe have been well documented and have been attributed to a number of stress factors (vanEngelsdorp and Meixner, 2010; Lee *et al*., 2015) that include pests and pathogens, pesticides and limited quality and availability of food resource (Klein *et al*., 2007; Neumann and Carreck, 2010; Potts *et al*., 2010; Becher *et al*., 2014; Goulson *et al*., 2015). These stressors interact with individual bees, resulting in lethal and sublethal effects that curtail longevity (Alaux *et al*., 2010; Aufauvre *et al*., 2012; Doublet *et al*., 2015; Retschnig *et al*., 2015) and alter fitness traits and behavioural and physiological performance, having implications for the entire colony (Becher *et al*., 2014; Rumkee *et al*., 2015). Pathogens affect behaviour directly through active manipulation evolved to facilitate transmission, although this is yet to be demonstrated in honey bees (see Mayack *et al*., 2015), and indirectly in response to an associated increase in the host's metabolic rate (Mayack and Naug, 2009; Naug and Gibbs, 2009; Mayack and Naug, 2015) or manipulating hormonal pathways (Mayack *et al*., 2015).

Although living in social groups has fitness benefits (Wilson, 1975), one of the trade‐offs is the increased risk of disease transmission because of close living quarters and high genetic relatedness (Schmid‐Hempel, 1998; Tarpy, 2003). Honey bee colonies comprise thousands of individuals living in close contact and predictably, pests and pathogens are wide‐spread and commonly occurring therein (Mouret *et al*., 2013; Manley *et al*., 2015; McMahon *et al*., 2015) and have been implicated in honey bee colony losses in the U.S. and Europe (Higes *et al*., 2008; vanEngelsdorp and Meixner, 2010). Deformed wing virus (DWV), Varroa destructor virus‐1 (VDV‐1) and *Nosema ceranae* (Fries) are three of the most prevalent pathogens present in European honey bee colonies (Martin‐Hernandez *et al*., 2007; Mouret *et al*., 2013; McMahon *et al*., 2015). The DWV complex (referred to henceforth as DWV) is a rapidly evolving and recombining group of closely related positive‐sense, single‐stranded RNA Iflaviruses, that includes VDV‐1 (de Miranda and Genersch, 2010; Moore *et al*., 2011; Zioni *et al*., 2011; Martin *et al*., 2012; Mordecai *et al*., 2016). DWV is vectored by the parasitic mite, *Varroa destructor* Anderson & Truman, (Martin *et al*., 2012) and is transmitted both horizontally (faecal–cannibal–oral) (Yue and Genersch, 2005; Mockel *et al*., 2011) and vertically (parent–offspring) (Chen *et al*., 2006; Yue *et al*., 2006; Yue *et al*., 2007; de Miranda and Fries, 2008; Yanez *et al*., 2012). Clinically relevant infections by DWV, defined as presence of DWV RNA in the brain (Mockel *et al*., 2011), do not always result in bees exhibiting a phenotype (deformed wings) (de Miranda and Genersch, 2010; Zioni *et al*., 2011). The microsporidian gut parasite, *N*. *ceranae*, historically a parasite of *A*. *cerana*, now includes *A. mellifera* as an alternative host (Higes *et al*., 2006) and causes no visible, external symptoms of infection. *N*. *ceranae* infects and reproduces inside epithelial cells of the midgut and is believed to be transmitted in the hive principally via the oral–oral pathway (Smith, 2012).

Prevalence and diversity of disease pathogens in honey bee colonies are probably greater than previously thought (Tentcheva *et al*., 2004; Siede *et al*., 2008; Furst *et al*., 2014; McMahon *et al*., 2015), since infection is often inapparent (Zioni *et al*., 2011; Mouret *et al*., 2013) or below the level of detection (Martin *et al*., 2013a). It is unsurprising, therefore, that research into the sublethal effects of commonly occurring pathogens on honey bee behaviour is limited, yet of increasing interest. Other than understanding transmission (Bowen‐Walker *et al*., 1999; Yanez *et al*., 2012; Manley *et al*., 2015), and influence on gene expression (Steinmann *et al*., 2015), physiology (Yang and Cox‐Foster, 2007) and learning (Iqbal and Mueller, 2007) of DWV, research into sublethal behavioural effects of pathogens has largely been limited to *N*. *ceranae*. This gut parasite has been shown to modify many aspects of honey bee behaviour, including increased maturation (Dussaubat *et al*., 2013; Goblirsch *et al*., 2013), impaired learning (Mallon *et al*., 2003; Kralj and Fuchs, 2010), enhanced energetic stress (Mayack and Naug, 2009, 2010; Mayack and Naug, 2015) and changes to flight and homing behaviour (Alaux *et al*., 2014; Naug, 2014; Wolf *et al*., 2014; Perry *et al*., 2015). *N*. *ceranae* has previously been shown to increase the number of foraging trips and flight duration, reduce the time spent in the hive (Alaux *et al*., 2014; Naug, 2014; Retschnig *et al*., 2015) and reduce foraging efficiency (Naug, 2014), but since individuals were not tracked once they had left the hive, the proportion of the time spent flying or resting was unknown. In contrast, exploring whether DWV affects flight behaviour in bees that do not exhibit the visual symptoms of deformed wings typical of high levels of infection (de Miranda and Genersch, 2010), yet may already be suffering altered physiological, neurological or immunological function remains to be done.

Flight performance of an individual can determine its potential resource‐gathering capability and in social insects, efficient resource acquisition can have profound effects at the colony level (Becher *et al*., 2014). The farther the distance and longer the duration an individual is able to travel allows more of the landscape to be exploited for resources. Understanding flight performance of foraging honey bees challenged by pathogens is therefore of key importance not only for effective colony management, but also for protecting pollination service provision (Potts *et al*., 2010) by managed and wild species. Measuring flight performance of individual bees is notoriously difficult though; bees are small, fast flyers covering vast foraging areas. Tracking individuals using harmonic radar (Riley *et al*., 1996) is currently the only technology available to record bee flight routes in the field, however, it is not possible to simultaneously track two or more individuals to estimate the spatial and temporal limits of bee flight under similar environmental conditions. In contrast, assessing flight performance using tethered individuals on flight mills provides an elegant opportunity to explore individual endurance limits allowing maximal control of environmental factors other than pathogen load (Brodschneider *et al*., 2009).

Here, we sought to test the null hypothesis that natural levels of infection by *N*. *ceranae* and the DWV complex (comprising DWV and VDV‐1) in forager honey bees have no effect on flight performance. Thus, the aims of this work were to (i) quantify the natural levels of infection by *N*. *ceranae* and DWV + VDV‐1 in actively foraging, apparently healthy honey bees and, (ii) elucidate the sublethal effects of these commonly occurring pathogens on flight distance and duration.

## Results and discussion

Of 127 bees that were analysed, 73 tested positive for one of the two pathogens that were screened, 20 tested positive for both pathogens and 34 tested negative for neither pathogen. DWV was more prevalent (83 bees) than *N*. *ceranae* (30 bees) and the level of co‐infection was lower (20 bees) than for single infection (*N*. *ceranae* 10 bees; DWV 63 bees). Of the bees that tested positive for DWV and *N. ceranae*, mean loads were 3.6 × 10^10^ ± SD 1.8 × 10^11^ copies head^−1^ and 1.7 × 10^4^ ± SD 2.1 × 10^4^ mid‐gut^−1^, respectively. The levels of infection we recorded are comparable to those reported elsewhere for standard, managed apiaries (Gauthier *et al*., 2007; McMahon *et al*., 2015; Steinmann *et al*., 2015).

Tethered flight mills have been successfully used to measure the relative flight performance of different taxa under controlled, standardised conditions (Riley *et al*., 1997; Blackmer *et al*., 2004; Spiewok and Schmolz, 2006; Brodschneider *et al*., 2009; Taylor *et al*., 2010; Dorhout *et al*., 2011; Jones *et al*., 2015), however it is important to recognise the limitations of this experimental technique. Flight mills restrict the physical and biophysical dynamics of natural flight, where reduced drag and a lack of need to produce uplift have been shown to result in lower levels of expended energy than are readily available (Riley *et al*., 1997) which could result in enhanced measures of flight performance than are possible when insects are in free flight. Equally, a lack of stimuli, such as olfactory cues from sources of forage, to initiate and sustain flight behaviour, could result in reduced measures of flight performance. Despite these differences to natural free‐flight conditions, tethered flight mills remain an important instrument for measuring the relative flight performance of worker honey bees, given the assumption that handling, tethering and restriction of natural cues affect the behaviour and performance of the test bees equally.

Contrary to our expectation and the findings of previous work (e.g. Alaux *et al*., 2014; Naug, 2014; Wolf *et al*., 2014), we found no effect of either presence of *N. ceranae* on flight distance (*F*_1, 121.5_ = 0.71, *P* = 0.400) or duration (*F*_1, 121.9_ = 1.39, *P* = 0.240) or the amount of *N*. *ceranae* on flight parameters (Table 1). Honey bees exclusively use carbohydrates to power flight activity (Sacktor, 1970; Rothe and Nachtigall, 1989) that accounts for 30% of the total energy expenditure of a forager bee (Harrison and Fewell, 2002). In this study, bees were flown to exhaustion before being fed a known and finite amount of energy in the sucrose meal (c.f. Gmeinbauer and Crailsheim, 1993; Hanauer‐Thieser and Nachtigall, 1995; Brodschneider *et al*., 2009) that fuels the subsequent test flight. The assumption is, therefore, that bees flown to exhaustion have no remaining energy reserves available to them (Gmeinbauer and Crailsheim, 1993). As an obligate gut parasite without mitochondria, *Nosema* species have been shown to cause energetic stress by reducing the amount of energy available to an infected bee. Trehalose, which is synthesised in invertebrate haemolymph from dietary sucrose and used for the rapid release of energy used in flight (Thompson, 2003), is decreased in bees naturally infected with *Nosema* and is thought to lead to significant decreases in flying ability (Mayack and Naug, 2010). In response to *Nosema*‐induced energetic stress, infected honey bees consume more energy‐rich food (Mayack and Naug, 2009; Martin‐Hernandez *et al*., 2011) and reduce food‐sharing with nest‐mates (Naug and Gibbs, 2009). Indeed, *Nosema*‐induced energetic limitations have been suggested as an underlying mechanism behind the increased likelihood of failure of foragers to return to the hive (Wolf *et al*., 2014), increased periods of time spent on foraging trips (Kralj and Fuchs, 2010; Alaux *et al*., 2014; Naug, 2014), and increased number of foraging trips (Dussaubat *et al*., 2013). However, the different experimental approaches of these studies may explain the apparent conflict in our results that naturally occurring, low levels of infection by *Nosema* have no effect on flight performance. Firstly, the studies did not directly measure flight duration and distance of individuals, rather they measured time spent outside the colony and were unable to distinguish between bee movement (flight) and resting. Secondly, the studies did not administer known quantities of energy prior to measuring flight activity, and so they were unable to determine the effects of *Nosema*‐induced energetic stress on honey bee flight. Thirdly, and perhaps most significantly, in some of the previous work, bees were inoculated with *Nosema* spores (Kralj and Fuchs, 2010; Dussaubat *et al*., 2013; Alaux *et al*., 2014; Naug, 2014; Wolf *et al*., 2014) that resulted in spore loads in the whole abdomen (Alaux *et al*., 2014) and mid‐gut (Dussaubat *et al*., 2013; Wolf *et al*., 2014) orders of magnitude greater than their controls, while the natural level of *Nosema* infection we report here was lower than the control groups (Dussaubat *et al*., 2013; Alaux *et al*., 2014). Finally, whilst we recognise that we tested for just two pathogens in our experiment, screening exclusively for *Nosema* in some previous work excluded other pathogens that may have influenced flight behaviour (Mayack and Naug, 2009; Naug and Gibbs, 2009; Kralj and Fuchs, 2010; Martin‐Hernandez *et al*., 2011; Dussaubat *et al*., 2013; Alaux *et al*., 2014).

**Table 1:** Results from intra‐block regression models fitting relationships between measures of honey bee flight performance and pathogen load, with pathogen effects fitted in different orders after accounting for flight mill differences (*N* = 93).

	Distance		Duration	
Model terms	*F*_1,86_	*P*	*F*_1,86_	*P*
Order 1				
+ DWV	9.07	0.003	8.81	0.004
*+ N*c	1.51	0.222	0.71	0.402
Order 2				
*+ Nc*	0.45	0.503	0.09	0.767
+ DWV	10.13	0.002	9.43	0.003

*Nc = Nosema ceranae*

Only 10 of the bees that were flown subsequently screened positive exclusively for *N*. *ceranae*, leading, retrospectively, to low statistical power (32%) for detecting differences in flight performance of *Nosema*‐infected and uninfected bees of the magnitude reported by Naug (2014) for inoculated bees. However, 99% confidence intervals for the observed ratios of flight durations for the two groups, based on either all bees flown or only on bees uninfected with DWV (see Supporting Information Appendix S1), did not contain values as extreme as the halving of flight duration for uninfected relative to *Nosema*‐infected bees that was reported by Naug (2014). Thus, we were unable to reject the null hypotheses of no statistical differences in flight performance between groups of bees uninfected and infected by low levels of *N. ceranae*, but we do not preclude or dismiss previously reported effects at higher levels of infection

**Figure 1:**
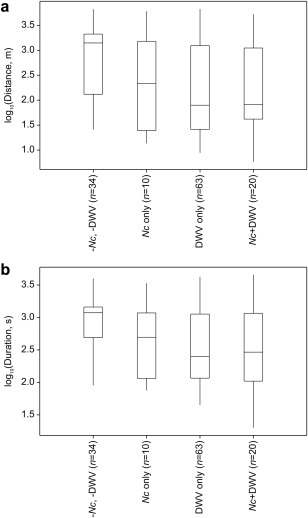
A. Box plots of log distance and B. log duration travelled by bees that tested negative for DWV and *N*. *ceranae* (‐pathogens), bees infected with *N*. *ceranae* only, DWV only and *N*. *ceranae* + DWV. Box: median (central line) ± quartiles; whiskers: minimum – maximum values. Number of bees tested in parentheses. Nc = *N*. *ceranae*.

Bees that were infected by DWV, and yet presented no obvious morphological symptoms of infection, flew shorter distances (*F*_1, 121.4_ = 10.17, *P* = 0.002) and durations (*F*_1, 117.1_ = 9.08, *P* = 0.003) than bees uninfected with DWV (Figs. 1A and B). Linear mixed modelling predicted bees infected and uninfected with DWV flew geometric mean distances of 150.0 m (95% confidence interval: 90.1–249.6 m) and 480.2 m (252.4–913.3 m), and durations of 347.1 s (255.6–471.3 s) and 718.3 s (480.2–1074.6 s) respectively.

DWV is transmitted to honey bees horizontally and vertically within the hive (Chen *et al*., 2006; Yue *et al*., 2006; Yue *et al*., 2007; de Miranda and Fries, 2008; Yanez *et al*., 2012) and is vectored by *V*. *destructor*, parasitising adults, larvae and pupae (Yang and Cox‐Foster, 2007; Gisder *et al*., 2009; Mockel *et al*., 2011). *V*. *destructor*, and by association, DWV, have been implicated in disrupting immunological responses and behaviour in asymptomatic honey bees. It has previously been shown that genes for protein repair and the labelling of protein for degradation were up‐regulated in pupae that were parasitized by *V*. *destructor*, while genes involved in wing development processes were down‐regulated (Navajas *et al*., 2008) suggesting disruption of larval and adult development. Furthermore, parasitism by the varroa mite of young adult worker bees with normal wings inhibited protein metabolism, energy production and expression of immune genes (Yang and Cox‐Foster, 2005; Alaux *et al*., 2011) and reduced longevity (Yang and Cox‐Foster, 2007). Significantly, studies have successfully linked varroa mite parasitism with the direct effects of infection by DWV, providing evidence for DWV‐induced reduced immune gene expression (Steinmann *et al*., 2015), impaired associative olfactory learning and memory formation (Iqbal and Mueller, 2007). Thus, it is clear that there are diverse effects of sublethal infection by DWV on honey bees. Our data suggest, for the first time, that DWV may affect another important behavioural function, in reducing flight performance. The mechanisms behind these reductions in flight duration and distance are, as yet unclear, but it is possible that the disruption in expression of genes associated with protein metabolism, energy production and internal wing development may reduce the physical fitness characters of the forager bees. Another explanation for reduced flight performance in bees infected with DWV may be related to pathogen‐induced accelerated behavioural development. Enhanced behavioural maturation from in‐hive to forager bees has been observed in bees infected with *N*. *ceranae* (Dussaubat *et al*., 2013; Goblirsch *et al*., 2013; Mayack *et al*., 2015) and reduced flight performance has been recorded for forager bees from colonies exposed to high levels of the DWV‐vector, *V*. *destructor* (Blanken *et al*., 2015). Blanken *et al*. (2015) found that increased body mass, a character associated with precocious foragers (Vance *et al*., 2009), partly explained the relationship between exposure to the varroa mites and reduced flight performance while Schippers *et al*. (2010) report differences in flight muscle biochemistry between polyethic groups that may explain poorer flight performance. McDonnell *et al*. (2013) note that the similarity in brain transcription profiles of control honey bees and those infected with DWV or *N*. *ceranae* suggest that any provocation of precocious foraging occurs because of self‐removal from the colony, as a form of social immunity (Meunier, 2015). We are unable to confirm an association between body mass, and thence DWV‐induced precocious foraging in our experiment, however our finding that there was no direct effect of DWV load on wing size (*F*_1, 17_ = 0.005, *P* = 0.945; *N* = 19), infers this may be a plausible explanation that warrants further investigation.

**Figure 2:**
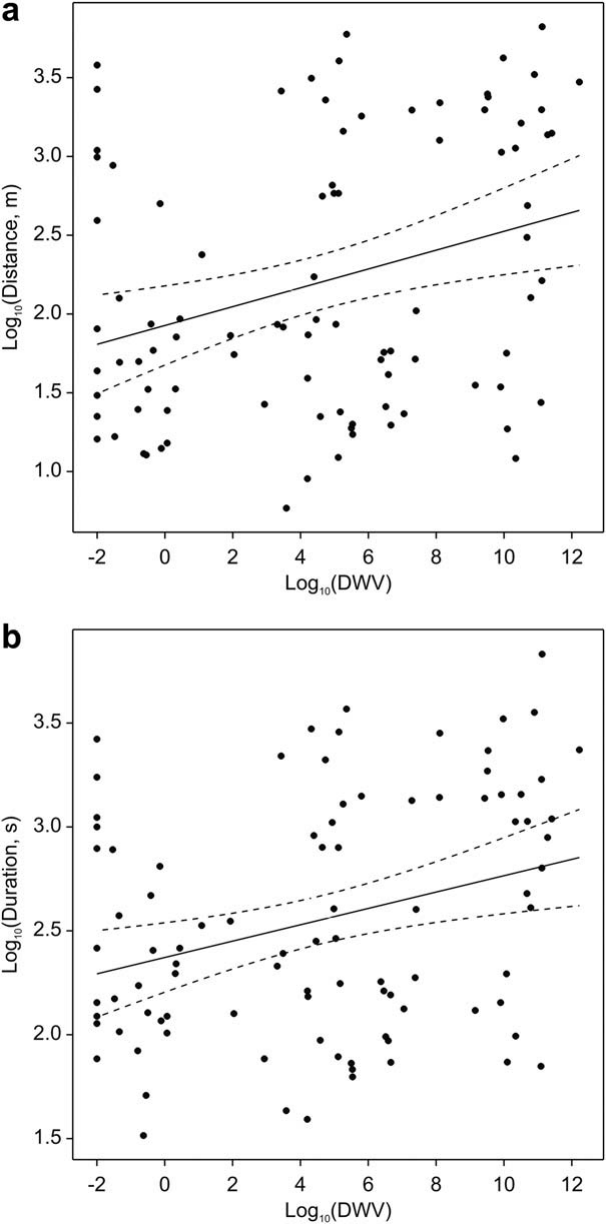
Fitted regression line (solid) with 95% confidence intervals (dashed) relating A. flight distance (regression coefficient = 0.060, SE 0.0199, *P* = 0.003) B. flight duration (regression coefficient = 0.039, SE 0.0132, *P* = 0.004) to DWV load of 93 bees flown on the flight mill.

Predictive models including only DWV indicate a weakly positive relationship between amount of DWV and flight distance (regression coefficient = 0.060, SE = 0.0199, *P* = 0.003) and duration (regression coefficient = 0.039, SE = 0.0132, *P* = 0.004) (Table 1). Whilst these relationships were significant, the models only accounted for 8.6% and 7.1% of the variation in distance and duration, respectively (Fig. 2A and B). This perplexing result requires further investigation, not least because so little of the variation was accounted for by DWV in the models, but also because the weak relationship over the range of levels of infection predicts such small increases in distance and time travelled (regression coefficients of 0.06 and 0.04), which cannot be considered to be biologically significant. Conducting dose–response assays of DWV and *N*. *ceranae* on flight behaviour and genomic response are prime areas of future research.

Co‐infection by the pathogens in this experiment occurred in 15.7% of the bees we tested and in agreement with Martin *et al*. (2013b), we found no association between the presence and absence of *N*. *ceranae* and DWV (Pearson *χ* ^2^ = 0.03, df = 1, *P* = 0.863) and nor were there interactions between the two pathogens and either distance (*F*_1, 121.6_ = 1.35, *P* = 0.248) or duration (*F*_1, 122.1_ = 1.08, *P* = 0.301). It is unlikely, then, that there were confounding effects of these pathogens on flight behaviour in this experiment.

In conclusion, the bees tested here were representative of colonies with natural levels of *N*. *ceranae* and DWV infection, where inapparent, but clinically relevant infection by DWV was shown to reduce the distance and duration of flights in forager bees. If the reduced flight abilities we recorded on the tethered flight mills operate in the field under natural, free‐flight conditions, it is likely that sublethal effects of DWV infection are more widespread than previously thought. Indeed, recorded (Genersch *et al*., 2010; Dainat *et al*., 2012) and predicted (Kielmanowicz *et al*., 2015) over‐winter colony losses have been attributed to natural levels of DWV infection. Possible consequences of reduced flight endurance per unit of energy include less efficient and more costly resource acquisition for the individual and for the colony, particularly in landscapes where forage resources are spatio‐temporally sparsely and patchily distributed, and enhanced risk of premature death because of increased exposure to predators and physiological fatigue. Predicting and scaling the implications of our findings under artificial conditions to colonies in natural field conditions where DWV is persistently and highly prevalent will require further studies to understand behavioural responses to pathogen‐mediated compromises in flight performance, including for example, whether there are trade‐offs between resource utilised by the bee and that contributed to the colony.

## Experimental procedures

### Honey bees

Returning, actively foraging honey bees with no visible signs of disease (dysentery or malformed wings) and carrying corbicular pollen were randomly selected on the morning of the flight test from five, conventionally managed apiaries, comprising 14 colonies, within 10 km of Rothamsted Research, UK (51°48′28.83″N, 000°22′31.58″W) in July, August and September 2012 and August and September 2013. The bees were placed into hoarding cages (Williams *et al*., 2013) with free access to 1M sucrose syrup and water and then placed in an incubator set at 32°C to allow preparation of the bees for flight performance testing on the flight mill.

### Flight performance

We recorded flight distance and duration as two measures of flight performance using roundabout flight mills, similar to those used in many studies to characterise insect flight ability (Bradley and Altizer, 2005; Brodschneider *et al*., 2009; Dorhout *et al*., 2011; Sappington and Burks, 2014). A set of five flight mills, that consisted of a lightweight arm suspended at the centre by two magnets forming an almost resistance‐free axis (see Chapman *et al*., 2015) and surrounded by equally sized and spaced monochrome vertical stripes surround each flight mill to provide the illusion of movement (Hrassnigg and Crailsheim, 1999), were located in a controlled environment room set at 24°C with constant overhead lighting. Test bees had an Opalith disc attached to the thorax (Human *et al*., 2013), before being allowed to rest in the hoarding cage for 45 minutes before being tested on the mill. Immediately prior to connecting to the flight mill, an attachment (15 mm x 1 mm) was glued (Evo‐Stik impact multipurpose adhesive) onto the Opalith disc. The bee, holding a small ball of paper between her legs as a stimulus for spontaneous flight (Brodschneider *et al*., 2009; Sappington and Burks, 2014), and complete with attachment, was then connected to the flight mill arm and a counter weight of similar mass was attached to the other end of the arm. The flight mill allows the bee to fly in a circular trajectory, with a circumference of 1m, and the embedded microcontroller board records the total distance and duration flown by the bee at 5 s intervals to the nearest 20 cm.

To control the amount of energy available for flight, each bee was allowed to fly to exhaustion in order to deplete the sugar reserves in the honey stomach prior to being fed a known amount of energy (Gmeinbauer and Crailsheim, 1993; Brodschneider *et al*., 2009). The exhaustion flight was completed when, despite being stimulated, the bee did not recommence flying for more than 30 s. The bee was removed from the mill, fed 10 µl of 1M sucrose solution using a pipette before being re‐attached to the mill for flight performance testing until the bee again ceased flying because of lack of energy. The bee was removed from the flight mill, placed in an Eppendorf tube and stored at −80°C. Test flights were terminated when, despite stimulation following a pause in flight, bees did not resume flight.

### Disease analysis

*Nosema* spore counts midgut^−1^ were determined microscopically using a Neubauer improved 5x5 haemocytometer` following the methods of Human *et al*. (2013). The digestive tract was removed from the bee, and the midgut was isolated and homogenised in 500 µl of distilled water using a micropestle. *Nosema* spores were counted in four haemocytometer chambers and the total number of spores in a volume of 0.28 mm^2^ was counted per chamber. To confirm species identification (*N*. *ceranae* or *N*. *apis*), spores were identified using species‐specific PCR (Fries *et al*., 2013; Wolf *et al*., 2014).

The presence of DWV‐complex RNA in the brains of honey bees with apparently normal wings indicates clinically relevant, overt infection by DWV (Genersch *et al*., 2010; Mockel *et al*., 2011), so we quantified absolute copy numbers of positive‐strand DWV and VDV‐1 in the heads of the test bees. Each head was homogenised in 600 µl of lysis buffer (RLT Buffer, Qiagen, Manchester, UK) with 1% β‐mercaptoethanol (Qiagen, Manchester, UK). RNA was extracted from this supernatant using the RNeasy Mini kit affinity column purification (Qiagen, Manchester, UK) in a QIAcube robot (Qiagen, Manchester, UK) and quantified using an Epoch microplate spectrophotometer (BioTek, Swindon, UK). Total cDNA was synthesized cDNA from 800 ng RNA using M‐MLV reverse transcriptase (Promega). For absolute quantification, duplicate qRT‐PCR was performed for each sample using SYBRgreen Sensimix (Bioline, Luckenwalde, Germany) in the following program: 5 min at 95°C, followed by 40 cycles of 10 s at 95°C, 30 s at 57°C, and 30 s at 72°C (read) and data were normalised using the honey bee reference gene RP49 (Lourenco *et al*., 2008). An RNA‐free HPLC‐water and a virus‐positive sample cDNA were run as negative and positive control respectively in each reaction run. Following PCR, DNA was denatured for 1 min at 95°C and cooled to 55°C for 1 min. A melting profile was generated from 55°C to 95°C (0–5°C s^−1^ increments). Absolute quantification of DWV and VDV‐1 was calculated using duplicate DNA standard curves of purified flanking PCR products with efficiencies between 90% and 100% and correlation coefficients (*R* ^2^) from 0.990 to 0.999. To account for potential variation in sample quality, an upper cycle threshold (Ct) of 35 was set for RP49, above which samples were not included in quantitative analysis (Blanchard *et al*., 2007; de Miranda *et al*., 2013). The primers used were: DWV: DWV‐F2, DWV‐R2a; VDV: VDV‐F2, VDV‐R2a (McMahon *et al*., 2015).

### Wing size

Acute DWV infection frequently results in, amongst other symptoms, malformed wings that render bees incapable of flight. In order to estimate effects of pathogen on wing size and subsequent flight performance, wings were removed from a subsample of the bees stored for pathogen analyses. Both forewings from 19 bees were mounted between two microscope slides and scanned (4800 dpi) before measuring the combined lengths of three wing venation characters as surrogate measures of wing size (D2, D3, D7, after Dedej and Nazzi, 1994; Jaffe and Moritz, 2010). Each character on both wings was measured three times, to account for measurement error, giving a mean total length of the characters per bee.

### Statistical analysis

The flights of 476 bees were assessed on the mills. Since we sought to test the effect of pathogens on the range of flight abilities of apparently healthy bees recorded on the flight mill, nonflyers (*N* = 31) were excluded from the analysis. Laboratory constraints necessitated the creation of a subset of individuals from the remaining 445 bees for subsequent disease analysis. Therefore, bees classified as strong or reluctant flyers (test flight duration greater or less than 10 min, respectively), were matched, as closely as possible, by test date and colony. This process created a pooled subset of 127 bees for which the effects of presence and amount of pathogen load on flight performance (Supporting Information Table S1) were analysed.

The disease variables were skewed and so were transformed to logarithms (base 10) after adding an offset of 0.01 to allow for the absence of pathogens. The measures of flight performance (distance and duration) were also logged (base 10) to achieve homogeneity of variances and normality.

We first tested whether disease status (based on a 2 × 2 factorial treatment structure representing presence and absence of each of the two pathogens) affects flight distance or duration using a linear mixed model fitted using restricted maximum‐likelihood (REML), with flight mill included as a random effect.

We then tested, for diseased bees only, the relationship between measures of flight performance and quantitative pathogen load, using an intra‐block regression approach (Welham *et al*., 2015). Initial analyses with calendar month included in the random model suggested negligible temporal effects hence the random model was subsequently simplified to include flight mill effects only. Block effects (flight mill) were fitted before pathogen terms, the latter fitted first individually, then together. Statistically nonsignificant fixed effects were dropped and the resulting parsimonious models were used to obtain predictions. Finally, we analysed the effect of DWV load on wing length (transformed to logarithms (base 10) after adding an offset of 0.01) using simple linear regression. All analyses were done using GenStat 17 (VSNI, 2014).

## Supporting information

Additional Supporting Information may be found in the online version of this article at the publisher's website:

**Table S1.** Bee disease, flight and wing data.

**Appendix S1.** Testing confidence intervals at 95% and 99% for predictions of flight durations of Nosema‐infected bees against previously reported data.
