# Supplementary material for: Flight performance of actively foraging honey bees is reduced by a common pathogen
Source: Environ Microbiol Rep. 2016 Jul 7;8(5):728–37. doi: 10.1111/1758-2229.12434 (PMC5091639; doi:10.1111/1758-2229.12434)
Supplement: Supplementary file 2 — Appendix S1. Testing confidence intervals at 95% and 99% for predictions of flight durations of Nosema‐infected bees against previously reported data. [file EMI4-8-728-s002.docx]

**Supporting Information Appendix 1**

**Confidence Intervals**

Confidence intervals (CIs) are given below for the observed ratios of mean flight durations (computed as the back-transformed differences between means on the log scale) using predictions from the linear mixed model analyses for both the (a) main effect of *Nosema*, and (b) the effect of *Nosema* in the absence of DWV. These give estimated ranges within which the true differences would be expected to lie 95% (or 99%) of the time. All the 95% and 99% CIs (see below) do not contain values as extreme as a halving of flight duration as reported in Naug (2014).

*(a) All bees (main effect means)*

Difference in predicted mean flight duration (log scale) for bees testing negative (*n* = 97) and positive (*n* = 30) for *Nosema*: 2.741 − 2.561 = 0.1796; SED = 0.1234; 122 df

Back-transformed difference in means (ratio) with 95% CI: (0.861, 1.512, 2.654)

Back-transformed difference in means (ratio) with 99% CI: (0.719, 1.512, 3.181)

(b) *Bees testing negative for DWV (interaction means)*

Difference in predicted mean flight duration (log scale) for bees testing negative for DWV and either negative (*n* = 34) or positive (*n* = 10) for *Nosema*: 2.927 − 2.619 = 0.3076; SED = 0.2010; 122 df

Back-transformed difference in means (ratio) with 95% CI: (0.812, 2.030, 5.076)

Back-transformed difference in means (ratio) with 99% CI: (0.605, 2.030, 6.818)

Naug, D. (2014) Infected honeybee foragers incur a higher loss in efficiency than in the rate of energetic gain. *Biology Letters* **10**.
